# The Aging Mitochondria

**DOI:** 10.3390/genes9010022

**Published:** 2018-01-09

**Authors:** Pierre Theurey, Paola Pizzo

**Affiliations:** 1 Department of Biomedical Sciences, University of Padova, Padova 35121, Italy; 2 Neuroscience Institute, National Research Council (CNR), Padova 35121, Italy

**Keywords:** aging, mitochondria, ROS, MFRTA, mitochondrial function, mtDNA mutations, mutator mouse, lifespan

## Abstract

Mitochondrial dysfunction is a central event in many pathologies and contributes as well to age-related processes. However, distinguishing between primary mitochondrial dysfunction driving aging and a secondary mitochondrial impairment resulting from other cell alterations remains challenging. Indeed, even though mitochondria undeniably play a crucial role in aging pathways at the cellular and organismal level, the original hypothesis in which mitochondrial dysfunction and production of free radicals represent the main driving force of cell degeneration has been strongly challenged. In this review, we will first describe mitochondrial dysfunctions observed in aged tissue, and how these features have been linked to mitochondrial reactive oxygen species (ROS)–mediated cell damage and mitochondrial DNA (mtDNA) mutations. We will also discuss the clues that led to consider mitochondria as the starting point in the aging process, and how recent research has showed that the mitochondria aging axis represents instead a more complex and multifactorial signaling pathway. New working hypothesis will be also presented in which mitochondria are considered at the center of a complex web of cell dysfunctions that eventually leads to cell senescence and death.

## 1. Introduction

On average, a healthy person lives 80 years and one of the highest risk factors known for most human diseases and mortality is aging. Despite the utmost importance of this observation, the origin and mechanism of aging remains unclear. To this regard, substantial advances have been made through investigations performed in cells and in vivo in lower organisms, such as *C. elegans* and *D. melanogaster*, as well as in mice. The former models offer extensive experimental advantages for these studies, i.e., shorter lifespan and easier genetic manipulation.

**Figure 1:**
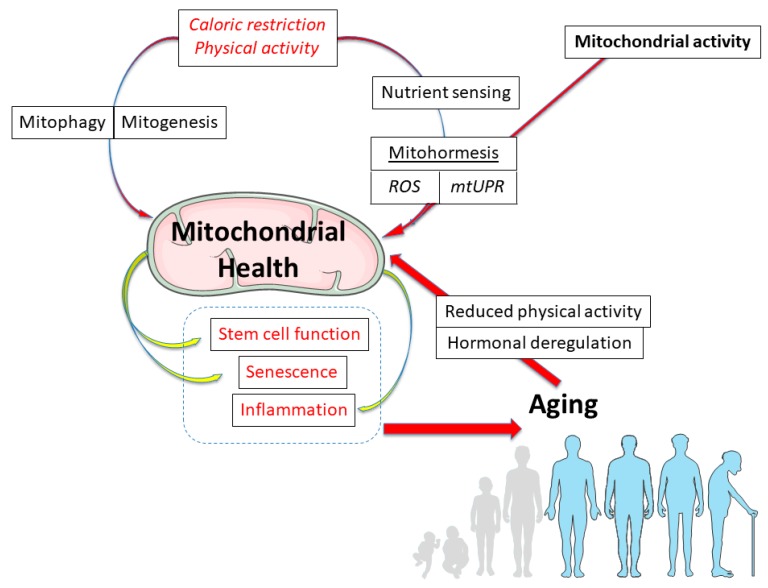
Mitochondrial Health at the center of a Cause–Consequence cell crossroad. The original, simplistic view of the Mitochondrial Free Radical Theory of Aging (MFRTA), postulating a mitochondrial activity/ROS/mtDNA isolated interaction, was progressively replaced by a more integrative view in which healthy mitochondria are the result of multiple cellular pathways and activities, impacting different aspects of aging, in diverse tissues and in different manners. See text for details.

Many evolutionary and mechanistic theories have been elaborated on, trying to explain why and how living organisms age. For instance, the “somatic mutation” theory suggests that aging is due to the increase other time in DNA damage and somatic mutation, while the “telomere loss” theory postulates a decline in cellular division capacity with age, linked to the progressive shortening of telomeres in somatic tissues. The “altered proteins and waste accumulation” theory rather advocates an accumulation over time of damaged proteins, protein turnover being essential to preserve cell function and the accumulation of altered proteins contributing to a range of age related disorders [1,2]. However, from a mechanistic point of view, among all the theories, those that see mitochondria as main actors occupy a particular place. Indeed, mitochondria have been at the center of one leading hypothesis for 50 years: the free radical theory [3]. Even though the scientific community has shifted to a more complex view to explain aging, embracing a network of events, mitochondria remain of high importance because of their central position in cell homeostasis of almost every tissue. Thus, as far as the description of molecular and cellular mechanisms are concerned, mitochondria have been shown to participate in every main aspect of aging: decline of stem cell functions, cellular senescence, “inflammaging” and many others [4,5] (Figure 1). The role of mitochondria in metabolic pathways is also particularly important. Their modulation, such as with caloric restriction and aerobic exercise, in turn, represents interventions than can increase lifespan [6,7] (Figure 1). Before any mechanistic consideration, however, the involvement of mitochondria in aging has been historically supported by several reports describing decreased mitochondrial functions in aged tissue [8].

## 2. Mitochondrial Dysfunction Is Associated with Aging

Mitochondrial alterations have been extensively described in aging tissues of many organs for a long time [9]. It has been particularly studied in muscle and heart [8] and sarcopenia and heart failure are two main causes of physical decline in the elderly. In particular, in these two tissues, but also in others like liver, brain and adipose tissue, mitochondrial alterations during aging are multiple. In particular, the number and density of mitochondria [10,11,12], as well as mitogenesis [13,14], have been showed to be reduced, whereas for mitochondrial dynamics and content contradictory inconclusive results have been reported [15,16] (see also [8] for a recent review). Importantly, mitochondrial function has been regularly reported to be impaired in different aging tissues, in terms of ATP production and respiratory chain (RC) capacity/activity [17,18,19,20]. 

A key reported feature of aging mitochondria was the increase in somatic point mutations and large deletions in the mitochondrial DNA (mtDNA) [21,22,23,24]. Interestingly, these mtDNA mutations have been shown to be responsible for mitochondrial dysfunction [23]. Since mtDNA is located very close to the major source of reactive oxygen species (ROS) [25], oxidative damages have been considered the main cause of mutations in mtDNA [26,27]. Indeed, the Mitochondrial Free Radical Theory of Aging (MFRTA) considers the oxidative damage of mtDNA as the primary event affecting RC proteins, inducing its dysfunction and increasing ROS production in a vicious cycle [3,28]. This theory was supported by a wealth of correlative data. Indeed, oxidative stress increases with age [29,30,31] and its reduction, by decreasing oxygen concentration or overexpressing in primary fibroblasts the extracellular or intracellular form of the copper-zinc (CuZn) antioxidant superoxide dismutase (SOD), increases cell lifespan and reduces senescence in vitro [32,33,34]. Furthermore, the downregulation of the same enzyme has been shown to induce cell senescence [35]. In vivo, intracellular CuZnSOD overexpression, together with catalase, increases the lifespan in drosophila [36]. An increase of longevity was also reported in mice upon the overexpression of the mitochondrial manganese SOD [37] or a catalase targeted to mitochondria [38]. Interestingly, overexpression of the extracellular form of CuZnSOD was shown to protect mice against aging-induced cognitive impairment [39] without increasing lifespan [40]. Missing the evaluation of mitochondrial function in these studies, the origin of the discrepancies between the results from intracellular and extracellular SOD remains unclear and further investigations would be required to solve the issue. Even though mitochondrial dysfunction has been clearly established in aging tissues, the majority of the studies remained exclusively correlative and, importantly, the observed RC decline could be secondary, for example, to hormonal alterations or reduced physical activity of the aging organism [41,42,43]. 

However, the MFRTA remained arguably the main theory for aging for many decades. Yet, this theory has been strongly challenged [44,45,46] and the scientific community had to adjust working hypothesis to fit with a more complex mitochondria-centered network of aging mechanisms. One of the key actors of this transition came from the creation of a fast-aging animal model, the “mutator” mouse. 

## 3. From the Mitochondrial Free Radical Theory of Aging to a More Complex View

The mutator mouse was created in 2004 by Nils-Göran Larsson’s group, to assess the role of mtDNA mutations and oxidative stress in aging [47]. Afterward, another group developed a very similar model with the same D257A mutation [48]. The mouse carries a homozygous knock-in mutation for a proof-reading deficient catalytic subunit of the mtDNA polymerase, leading to an extensive increase in point mutations and deletions in mtDNA. Importantly, the mouse displays a reduced lifespan and premature onset of aging-related phenotypes, providing for the first time a causative link between mtDNA mutations and aged phenotypes [47]. Moreover, experimental evidence indicated that the mouse phenotypes, typical of progeria (a pathology characterized by an extremely fast aging), were driven by mtDNA alterations [48,49,50]. 

The mutator mouse, however, did not display any increased oxidative stress [48,51] even though the animal clearly exhibited substantial mitochondrial dysfunction [50,51], shedding doubts on a direct link between mtDNA mutations, ROS and mitochondrial alterations. Similarly, the N-terminal methyltransferase 1 (NRMT1) knockout mouse exhibits decreased mitochondrial function and phenotypes of premature aging, despite reduced ROS generation [52]. In addition, a study reported a lack of gain in longevity in mice overexpressing the mitochondrial manganese SOD (MnSOD), despite decreased oxidative stress and age-related decline in mitochondrial ATP production [53]. Moreover, further genetic investigations in aged individuals showed that the evolution rate of the mitochondrial mutation load was incompatible with a ROS-mediated mechanism that would predict a mutational burst not observed experimentally [7,54]. Instead, recent works suggested that de novo mutations come rather from DNA polymerase errors [55,56], or because of clonal expansion of original maternally inherited mutations [7,57,58]. The role of mtDNA mutations in the mutator mouse was furthermore challenged by different observations: (i) mtDNA mutations were order of magnitudes higher in the model animal than in aged human tissues [59]; (ii) the mutation load of corresponding control normal-aging mice were still higher than that in aged humans [60]; (iii) the heterozygous knock-in mutator mouse exhibited a major increase in mutation load, compared to that of aged wild type mice, with no phenotype of premature aging [61]. Additionally, concerns have arisen regarding the type of mutations accumulated in the mutator mouse, which did not include conical mtDNA deletion observed in humans [62]. Another important challenge to the mtDNA mutation-mediated theory of aging came from another mouse model. Indeed, the “deleter” mouse, which expresses a mutant-dominant version of a mitochondrial replicative helicase, exhibits accumulation of large-scale mtDNA deletions and mitochondrial dysfunctions, leading to mitochondrial myopathy without displaying premature aging phenotype [63]. 

All these findings found echo in studies questioning whether somatic mutations in mtDNA could ever reach a level high enough to have a significant physiological impact on mitochondrial function [63]. Indeed, among the natural mtDNA heteroplasmy, a pathogenic mutation would need to rise up from 60% to over 95% of level to have a functional impact on the RC [64,65,66]. This phenomenon, called mitochondrial threshold, led many scientists to doubt the real implication of mtDNA mutations in the aging process. However, an alternative hypothesis of a random mitotic segregation of mutated mtDNA, called relaxed replication of mtDNA [67], would implicate the formation of a mosaic cell distribution of mitochondrial deficiency in the aged tissue, which would actually fall in line with many experimental observations [23,56,68]. 

Recently, it has been demonstrated that the progeria-like phenotype of the mutator mouse derives from a somatic stem cells (SSCs) dysfunction, leading to defects in self-renewal of old post-mitotic tissues [69]. The SSCs defect is already present at the embryonic state and precedes RC malfunctioning, which arises only when the aging phenotype is already present in the mouse, strongly suggesting that the SSCs dysfunction represents an early event in the mechanisms leading to aged phenotypes. 

## 4. A Complex and Mitochondria-Centered View of Cell Dysfunctions in Aging

One of the main topics of investigation in the aging field is the link between SSCs dysfunction and mitochondrial decline in aged tissue [4]. Indeed, stem cell exhaustion is a phenomenon that has been described in many tissues as part of the aging process [70,71,72]. One of the first indications of the role played by mitochondria in the SSCs decline was the observation that in the mutator mouse SSCs dysfunctions could be partially reversed by antioxidants [69]. Moreover, the efficiency of somatic cells reprograming into induced pluripotent stem cells (iPSC) was severely impaired in the animal, in a ROS signaling-dependent manner [73]. Interestingly, the SSCs decline is relying on point mutation accumulation in their mtDNA since the deletor mouse model, which displays mtDNA deletion only in post-mitotic tissues, does not display SSCs renewal impairment [69]. These observations fall in line with the idea that, in general, mitochondria are important in the metabolic control of stem cells pluripotency through specific mitochondrial metabolites, such as α-ketoglurate [74] or NAD+ [75]. The importance of mitochondria in maintaining stem cells functions could also be suggested by the fact that these cells have a unique capacity to eliminate old mitochondria through increased mitophagy [76] and specific segregation of old mitochondria [77]. However, it is important to keep in mind that SSCs decline alone does not recapitulate all the features of physiological aging [78], and that mitochondria can affect many other related cellular phenomena, such as senescence. 

Indeed, as for stem cells differentiation, senescence is characterized by profound metabolic changes [79] and induction or repression of senescence goes through metabolic regulation. For example, though the context might be different from aging, cell senescence mediated by oncogenes, such as *BRAF* and *p53*, involves the modulation of mitochondrial enzymes and metabolism [80]. Other interesting elements for understanding the complex relationship between aging and mitochondrial metabolism come from yeasts, where it has been shown that the overexpression of the mitochondrial enzyme malate dehydrogenase increases lifespan [81,82]. Moreover, from large scale screenings, many genes coding for tricarboxylic acid cycle (TCA) enzymes have been identified as potential regulators of yeast replicative lifespan [83]. These findings are supported by the ability of malate and fumarate to extend lifespan in *C. elegans* [84], despite the lack of experimental evidence in mouse models. Mitochondrial metabolism is also a key factor in nutrient sensing (NS) mechanisms [85] that are crucial regulators of lifespan. Indeed, the ability of caloric restriction (CR) to increase lifespan has been well established and this latter effect has been shown to go through mitochondrial metabolism-mediated NS. Many studies in yeast, and in a wide range of multicellular lower and higher organisms, have shown that CR simultaneously increases lifespan while improving mitochondrial activity [86,87,88] and mitochondrial biogenesis [89], with peroxisome proliferator-activated receptor gamma coactivator *1*-*alpha* (PGC-lα) and sirtuin 1 (SIRT1) being the two main regulators of the process identified so far [90,91]. Interestingly, the inhibition of NS signaling pathways, such as the Insulin/IGF-1 [92] and mechanistic target of rapamycin (mTOR) [93,94] pathways, led to similar results. Moreover, it was further showed that ROS signaling and adenosine monophosphate (AMP)-activated protein kinase (AMPK) activation could be the common mechanism linking CR, inhibition of NS pathways and mitochondrial activity [95,96]. 

The view of ROS as signaling molecules in the cellular antioxidant pathway, rather than deleterious byproducts, led to the concept of mitochondrial hormesis (mitohormesis), corresponding to the idea that an increase in ROS production can eventually induce an adaptive response that will overcome the increase of oxidative stress and, eventually, has beneficiary effects [97]. This is particularly true during aerobic exercise, another key intervention than can positively influence aging, which increases ROS production while simultaneously improving mitochondrial biogenesis, function, and metabolic health in the elderly [98,99,100]. Similarly, the link between mitochondrial function and lifespan is complex, and cannot be oversimplified to the idea that highly active mitochondria increase lifespan [89]. Indeed, studies have demonstrated that mild reduction of mitochondrial function can counter-intuitively increase lifespan in yeast, worms, flies and mice. In particular, this phenomenon was extensively studied in *C. elegans*, in which genetic manipulation of genes encoding RC components, to induce mild mitochondrial dysfunction, underlies some lifespan-extending programs [101,102,103]. This line of evidence has been further supported in different long-lived mouse models [104]. This opposite association of mitochondrial activity and lifespan was shown to involve compensatory mechanisms related to ROS signaling [105] and the mitochondrial unfolded protein response (UPR^mt^) [102,106]. The UPR^mt^ is an adaptive stress response to depletion in mtDNA or accumulation of unfolded proteins in the mitochondria that triggers a complete adaptive response including a nucleus-mitochondria and inter-organs cross talk for the induction of chaperone protein expression [107], ROS defense and metabolic adaptation. The UPR^mt^ is the proof of the utmost importance of mitochondrial homeostasis for cell health since more than 400 genes are involved to activate numerous maintenance pathways in the case of mitochondrial distress, to stabilize and recover their function. A subtle equilibrium constitutes a vast mitochondrial surveillance system to monitor the health of the mitochondrial pool [108]. However, the relationship between mitochondrial dysfunction, UPR^mt^ and longevity is complex, as it appears that UPR^mt^ activation, alongside multiple pathways activated during mitochondrial dysfunction, is required but not sufficient for gaining longevity [108,109]. Another major mechanism in maintaining mitochondrial protein homeostasis is mitoproteases, proteolytic mitochondrial enzymes important in protein quality control and in preventing accumulation of misfolded proteins. Interestingly, mitoproteases overexpression increases lifespan, even though these experiments were performed only in a fungal model of aging [110,111]. 

When the UPR^mt^ is overwhelmed, coordination of mitogenesis and mitophagy, which is the selective removal of damaged mitochondria by autophagy, ensures a fresh and functional pool of mitochondria sustaining cellular function [112]. Even though Parkin (a key protein in mitophagy; [113]) overexpression or suppression, respectively, increase or decrease lifespan in drosophila [114,115], direct evidence that mitophagy can modulate aging in other organisms is missing [4]. However, some clues indicate that mitophagy can modulate the effect of lifespan-controlling pathways. For example, it has been reported that the knockdown of mitophagy-related proteins decreases lifespan of mutant or CR long-lived *C. elegans* [116,117]. Moreover, Parkin overexpression attenuates aging-related muscle atrophy in mice [118], while its ablation decreases lifespan [119].

Thus, it appears that maintaining healthy mitochondria, not necessary active, is the central elements for healthy aging and long lifespan. The utmost importance of preserving the integrity of the mitochondrial pool is also sustained by the fact that mitochondria-derived damage-associated molecular patterns (DAMPs), in particular free mtDNA molecules and formylated peptides, liberated upon physical damage to mitochondria, can induce inflammatory response through multiple pathways [120,121,122]. Importantly, chronic inflammatory state is one of the hallmark of aging and has been termed “inflammaging” [123]. This process directly participates to the physical decline in the elderly, as illustrated by the fact that, in mice, ablation of the Nlrp3 inflammasome protects against age-related pathologies [124]. Moreover, mitochondria have been shown to mechanistically participate to the antiviral immune response: indeed, the innate immunity adaptor protein MAVS has been shown to be localized at the mitochondrial outer membrane [125]. Altogether, these findings strongly support the idea of an intimate relationship between mitochondria and the inflammatory process, in which the activation of this latter induces mitochondrial impairment and vice versa. Accordingly, many inflammatory pathologies have been associated to mitochondrial defects [126,127]. The importance of mitochondrial health in inflammation, one of the main biological response in maintaining organism homeostasis, is another example of the key role played by this organelle in the eukaryotic cell in general, beyond aging pathways. 

## 5. Conclusions

Human lifespan has increased drastically in the past decades and is likely to keep raising [127,128]. Increasing number of people at old and very old ages will pose major challenges for healthcare systems and, in this context, the promotion of healthy aging is crucial. To this regard, the better knowledge of the molecular mechanisms behind the aging process is a main priority of modern societies. Unfortunately, they are still unclear and the current working models are of increasing complexity [129]. In the past and in the current hypotheses, mitochondria had, and still have, a key position due to their central role in eukaryotic cells/tissues, in almost every form of homeostasis (Figure 1). Even though mitochondrial impairment is clearly associated with aging, the high complexity of aging phenotypes, and their underlying molecular mechanisms, make the deciphering of the real causing elements difficult [5]. Moreover, the discovery of mitohormesis in stress response and ROS signaling pathways nuanced the idea of active and healthy mitochondria, and of ROS production and oxidative stress. Indeed, as described above, increased ROS and less active mitochondria can promote healthy aging and long lifespan. Even in this complex scenario, more than ever, acting on mitochondria seems to be an attractive perspective in order to achieve gain in health and lifespan, since rejuvenating aged mitochondria could be an interesting therapeutic strategy to improve health in the elderly (Figure 1). To this regard, the best interventions identified so far in mammals and different aging models remain dietary intervention [130,131,132] (i.e., caloric restriction, intermittent feeding, nutrient deprivation or other fasting-mimicking diets) and physical activity [133].
